# Accurate Detection and Localization of Water Pipe Leaks through Model-Based TDR Inversion

**DOI:** 10.3390/s23020710

**Published:** 2023-01-08

**Authors:** Marco Scarpetta, Andrea Cataldo, Maurizio Spadavecchia, Emanuele Piuzzi, Antonio Masciullo, Nicola Giaquinto

**Affiliations:** 1 Department of Electrical and Information Engineering, Polytechnic University of Bari, Via E. Orabona 4, 70125 Bari, Italy; 2 Department of Engineering for Innovation, Complesso Ecotekne-Corpo O, University of Salento, 73100 Lecce, Italy; 3 Department of Information Engineering, Electronics and Telecommunications (DIET), Sapienza University of Rome, 00184 Rome, Italy

**Keywords:** leak detection, pipeline inspection, time domain reflectometry, TDR inversion, spatial TDR, water leakage, model-based measurements

## Abstract

The problem of water scarcity affects many areas of the world due to water mismanagement and overconsumption and, more recently, to climate change. Monitoring the integrity of distribution systems is, therefore, increasingly important to avoid the waste of clean water. This paper presents a new signal processing technique for enhancing the performance of the methodology of leak detection in water distribution pipes based on time domain reflectometry (TDR). The new technique is based on a particular kind of TDR inversion (spatial TDR) based on a “gray-box” lumped parameter model of the system. The model does not include, e.g., radiative phenomena, non-TEM (transverse electromagnetic) modes etc. but is capable of reproducing accurately the complicated reflectograms obtained by a TDR leak detection system assuming a proper profile of capacitance per unit length along the sensing element. Even more importantly, the model is identified using only the reflectograms taken by the system with very little prior information about the system components. The developed technique is able to estimate with good accuracy, from reflectograms with unclear or ambiguous interpretation, the position and the extension of a region where water is located. The measurement is obtained without prior electromagnetic characterization of the TDR system components and without the need of modeling or quantifying a number of electromagnetic effects typical of on-site measurements.

## 1. Introduction

Pipeline leakage is a significant problem in the public water supply industry. According to EurEau (European Federation of National Associations of Water Services), the mean leakage rate of drinking water distribution networks throughout Europe is about 25% of all water provided [1]. This means that approximately 2700 m^3^ of water are wasted yearly per km of pipe. Financial losses for suppliers are the most direct consequences of water waste due to leaks, but this problem has environmental and social consequences too. Leaks can indeed exacerbate the water scarcity issue, which, at certain times of year, is affecting a growing number of people and bigger areas of Europe as a result of climate change [2]. For these reasons, the problem of water leakage has been addressed by a recent European Union (EU) Directive [3], which states that water leakage levels should be monitored and reduced. Reliable and accurate methods for locating leaks in pipes are, therefore, of great interest nowadays.

Many different approaches have been proposed and developed over the years for leak detection in water pipes [4]. The most direct method is based on visual inspection, which relies on the usage of endoscopes or robots equipped with cameras and guided inside pipes. This is a very simple method conceptually, but it also requires a big specialized human intervention for interpreting images and data. Electromagnetic (EM) inspection methods such as magnetic flux leakage, remote field eddy current and ground penetrating radar are also used for status assessment of metallic pipes. These techniques evaluate the metal conditions based on the interaction between transmitted EM signals and the metallic pipes. Other methods are, instead, based on the use of acoustic and ultrasonic waves [5]. The effectiveness of all these methods depends on the kind of material and on the geometry of the pipes, on the working conditions of the water distribution network (e.g., water pressure) and on the environmental conditions. Even more importantly, expert personnel are required for measurement operations and for result interpretation, which prevents them from being considered for continuous monitoring of pipes.

**Figure 1:**
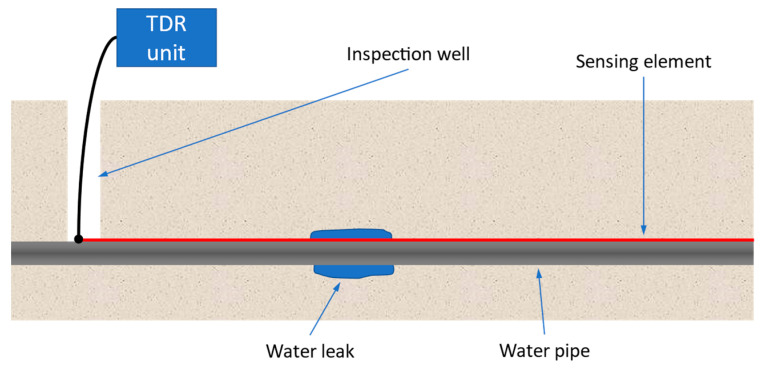
Representation of the on-field measurement setup for the leak localization in underground pipes based on time domain reflectometry (TDR). The sensing element (SE) is placed in the ground near the water pipe and is connected to a TDR unit through a short coaxial cable.

An alternative approach proposed in 2012 [6,7,8] is based on the use of time domain reflectometry (TDR). The method exploits EM waves propagating in a distributed sensing element (SE) placed near the pipe (typically less than 1 cm from the pipe) to detect and localize water leaks, as shown in Figure 1. The SE is a transmission line (TL), e.g., a bi-wire cable, whose primary parameters are affected by changes in the dielectric constant of the surrounding medium. The presence of water leaking from the pipe increases locally the dielectric constant of the medium and can, therefore, be detected by a reflectogram, as a reflection caused by the locally increased capacitance per unit length. The reflectogram is obtained by transmitting a stimulus signal from one end of the SE and measuring the reflected signal at the same point. In this way, long segments of the pipe can be analyzed with just one measurement. As a matter of fact, SEs up to 100 m long have been successfully used for water leak detection [6]. As of the current date, systems of this kind have been installed in more than 50 km of water pipes by AQP (public water operator of the Apulia region, Italy, and the largest water facility in Europe), proving the effectiveness of the methodology.

With respect to other available techniques for water leak detection in pipes, the TDR-based method has the advantage of not imposing requirements on the construction material of the pipes (because water in the soil around the pipe is detected) or on their operating conditions, leading to greater ease of use. TDR-based techniques also drastically reduce the amount of time needed for inspection compared to conventional leak detection techniques; in fact, hundreds-of-meters-long pipes can be inspected with a single measurement operation. Besides, TDR is a well-established technique already used for many applications such as detection and localization of cable defects [9,10,11,12], monitoring of landslide [13,14], characterization of antennas [15] and measurement of dielectric properties of materials [16,17], of liquid level in tanks [18], of soil moisture [19], of water diffusion in irrigation [20] among many others, even in the biomedical field [21]. A possible disadvantage of TDR-based water leak detection is that the SE must be installed near the water pipe, and, hence, it is difficult to use the technique in existing pipelines. This is not very limiting, however, since the SE can be installed during usual maintenance operations, which provide for the periodic replacement of sections of pipes.

A limitation of the TDR method is that the local increase in the dielectric constant caused by the leak must be clearly visible in the reflectogram in terms of a clear difference [7] between the “reference reflectogram” (taken without leak), and the “measurement reflectogram” (taken in presence of leak). As better shown in the next sections, this is not always the case: quite often, an incipient water leak causes a comparatively small variation of dielectric constant, which cannot be accurately detected visually, since the corresponding variation in the reflectogram is small or masked by other disturbance factors and unwanted effects which typically influence on-field measurement. It is worth noting that, in this case, evaluating a simple “difference reflectogram” between the measurement and the reference reflectogram is not a solution: a local change of dielectric constant causes changes in the whole reflectogram due to reflections, changes in the propagation velocity, etc. This is clearly observable in the examples reported in Section 3.

As a matter of fact, when the measurement reflectogram is not clearly interpretable, a *TDR inversion*–or spatial TDR–is a key issue for localizing, more accurately than an intuitive visual interpretation, the change in the spatial profile of dielectric constant. This operation is also useful for an accurate *localization* in addition to a reliable *detection.* Indeed, it must be noted that having a reasonably accurate measurement of the leak position is very important for the repair team, who is required to carry out excavations.

TDR inversion algorithms have already been presented and used successfully in the literature but in experimental situations quite different from the one here considered. Usually, the inversion problem is reduced to the minimization of an objective function whose gradient has a known analytical expression. In [22] and [23], the TDR inversion works well but for simulated ideal models. In [24], the TDR inversion is performed for an experimental setup, which uses a soil moisture TDR probe with accurately known electrical parameters (a classic flat-ribbon cable with three conductors) and in conditions allowing the use of a very simple model of its primary parameters. The same kind of TDR probe is used in [25], and, in this case too, the electrical parameters of the probe are accurately determined and validated through laboratory experiments and full-wave simulations. A satisfactory TDR inversion is also obtained in [26]: in this case, the probe is carefully characterized with frequency domain measurements, performed using a properly calibrated VNA (Vector Network Analyzer). In general, in all TDR inversions known to the authors, determining the parameters of the employed probe and of the other components of the measurement system is a preliminary step, necessary to performing the actual inversion.

In the set-up depicted in Figure 1, an accurate characterization of the measurement system is unavailable and cannot be obtained a priori with, e.g., VNA measurements. First of all, the probe, i.e., the SE, is a buried bi-wire cable. Therefore, it is tens of meters long, inevitably twisted and stressed, and it is surrounded by a medium with an uneven and unknown profile of dielectric constant. Characterizing a short section of the SE in air is not simple (it is a balanced line, not matched with the ports of a VNA), but, more importantly, the characterization would be of no use to model the cable when it is buried near the pipe.

In the experimental situation of Figure 1, other parameters are also not known with sufficient accuracy: namely, the electromagnetic parameters of the coaxial cable and of its connection with the TDR unit are not necessarily the same that could be determined in laboratory making use of dedicated instrumentation. These parameters should be determined for the actual setup on the field. The same applies, of course, to the SE termination.

Consequently, classic TDR inversion methods are substantially inapplicable to the problem at hand: a method that does not require prior characterization of the measurement system components is needed. It must also be underlined that classic methods often use reflectograms taken from both ends of the probe (in early works like [22,23], and in recent works like [27,28]) since TDR inversion with a single reflectogram, under the hypotheses considered in these papers, is mathematically an ill-posed problem. Instead, a TDR inversion with a single reflectogram, like in [29], is highly desirable for the considered problem of water-leak detection, but it is necessary not to limit too severely the class of identifiable capacitance profiles.

In this paper, a TDR inversion method suitable for water-leak localization in underground pipes, using a setup similar to that described in Figure 1, is proposed. The basic idea is to identify a circuital model of the on-field measurement system, able to reproduce very accurately both the “reference reflectogram” and the “measurement reflectogram” even if:

(1) the reflectograms are very irregular, a condition inherent to the situation of a long SE buried along a pipe;
(2) the electromagnetic parameters of the SE, and of other components of the measurement system, are not known in advance, and must be determined directly on the field.

This model is not designed to represent accurately the physical reality of the system (which includes non-TEM (transverse electromagnetic) modes, radiative phenomena, interfering fields, non-ideality of connections, etc.) but simply to reproduce the observed reflectograms including the multiple reflections (voltage recorded after the first reflected pulse).

The circuital model, and the identification procedure, are then used for leak detection and localization, based on measuring the capacitance profile needed to reproduce the reflectogram as it derives from the presence of a leak. As shown in the next sections, the proposed technique has clear advantages with respect to traditional techniques when analyzing reflectograms measured in the presence of water leaks which produce small variations of dielectric constant. In these cases, indeed, results obtained with traditional techniques based on the observation of the reflectogram are very ambiguous while the proposed algorithm is able to effectively detect and localize the water leak.

It is worth noting that the proposed method can be easily extended to a number of other TDR-based applications, such as those for detecting soil moisture, cable faults, defects in transmission lines, etc. The advantage of the method is that almost no prior knowledge of the electrical parameters of the probes and of the measurement system components is required, which makes it more flexible and general, and much easier to apply. Investigating such different application of the method, of course, is the subject for separate works.

## 2. Materials and Methods

### 2.1. Reflectograms in TDR Leaks Detection in Water Pipes

The on-field measurement setup for TDR-based leak localization in underground water pipes (Figure 1) has been already illustrated and discussed with some detail in the Introduction. We add here that the TDR unit can be a dedicated commercial device or can be realized with general purpose instruments (e.g., waveform generator + oscilloscope).

**Figure 2:**
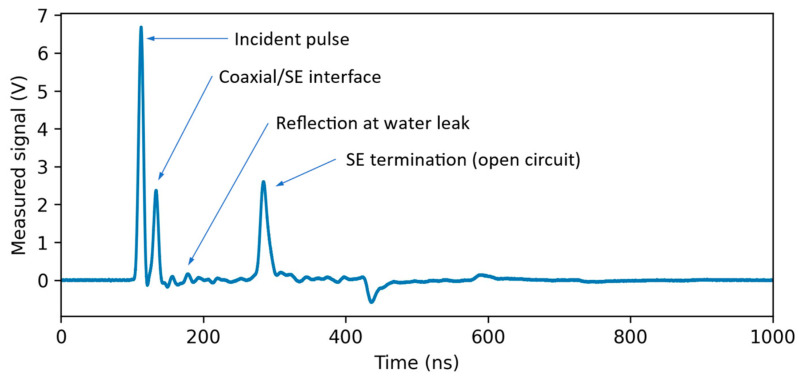
Typical reflectogram associated to the on-field measurement setup schematized in Figure 1, when an impulsive transmitted signal is used by the TDR unit.

A typical signal obtained using the described measurement setup, with the SE terminated in open circuit, is shown in Figure 2. An impulsive voltage waveform is transmitted to the SE and reflected at different points. The first significant reflection occurs at the interface between the coaxial cable and the SE and is due to the quite different characteristic impedance of the connected TLs. The reflection at the SE end is also very strong, due to the open termination. The reflection at the water leak is also visible in the example, but, in this case, its amplitude is comparable to other small reflections due to non-idealities of the SE (e.g., twists and stress on the bi-wire) and to the inhomogeneity of the medium around it. In this TDR signal, the water leak is hardly detectable and localizable. Detection and localization in this situation is exactly the goal of the method illustrated in this work.

### 2.2. Laboratory Experimental Setup

**Figure 3:**
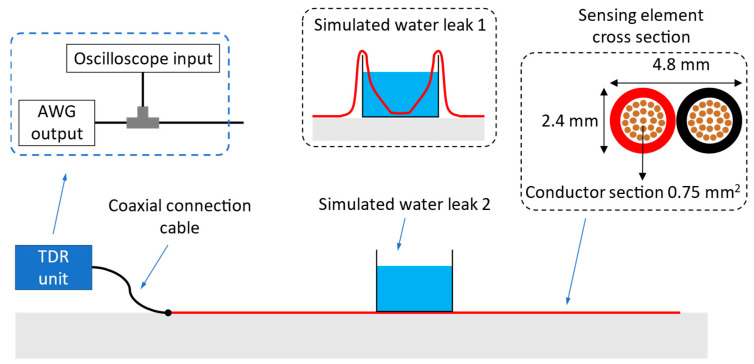
Representation of the laboratory measurement setup. The SE is placed on the floor and is connected to the TDR unit through a short coaxial cable.

For the purpose of the present investigation, an “ad-hoc” laboratory set-up has been arranged, described in Figure 3. The TDR unit is implemented using an Agilent 33250A arbitrary waveform generator (AWG) for the stimulus signal and a LeCroy Waverunner-2 LT262 oscilloscope for signal acquisition. The AWG and the SE are connected to the oscilloscope through a T-junction and a short RG58-CU coaxial cable. The SE is a bi-wire cable having the cross-section shown in Figure 3. The two conductors of the SE are composed of 24 copper-clad aluminum wires coated with polyvinyl chloride (PVC).

The whole setup is designed to perform several laboratory experiments with many different accurately known distributions of water near the SE. Nonetheless, it is not so easy to obtain an accurate electromagnetic model of the system, and this situation mimics an on-field measurement setup.

First of all, the SE is 15 m long and lies freely on the floor: it is, therefore, unavoidably twisted at random points. The parameters of the TL constituted by the SE are influenced by the floor and are not the same as would be observed in air. The SE is not shielded from electromagnetic fields in the laboratory. The connection between the (unbalanced) coaxial cable and the (balanced) SE is realized with simple crocodile clips, and, therefore, its effect on the reflectogram, although certainly not very large, is not known.

It is useful to note that, in practical on-field leak measurements, the SE has more distance between the wires and, therefore, higher sensitivity. In laboratory experiments it is preferable to use an SE with low sensitivity, since it makes it simpler to obtain realistic reflections associated with leaks using only water, instead of a mix of soil and water.

More specifically, in our experiments water leaks are simulated by (method 1) submerging the SE in water or (method 2) by placing a water container on the SE. Method 1 produces a visible reflection, like those observed in the field with a more sensitive SE, when a big leak soaks the soil surrounding it. Method 2 produces a much weaker reflection, like those observed in the field when the leak is smaller and the surrounding soil is moist but not soaked. It is worth noting that the placement of the water container above the SE is a practical device to obtain a weaker reflection in laboratory tests and does not replicate an actual on-field condition. In installed systems, indeed, all the soil around the SE is moist due to water leaking from the pipe.

**Figure 4:**
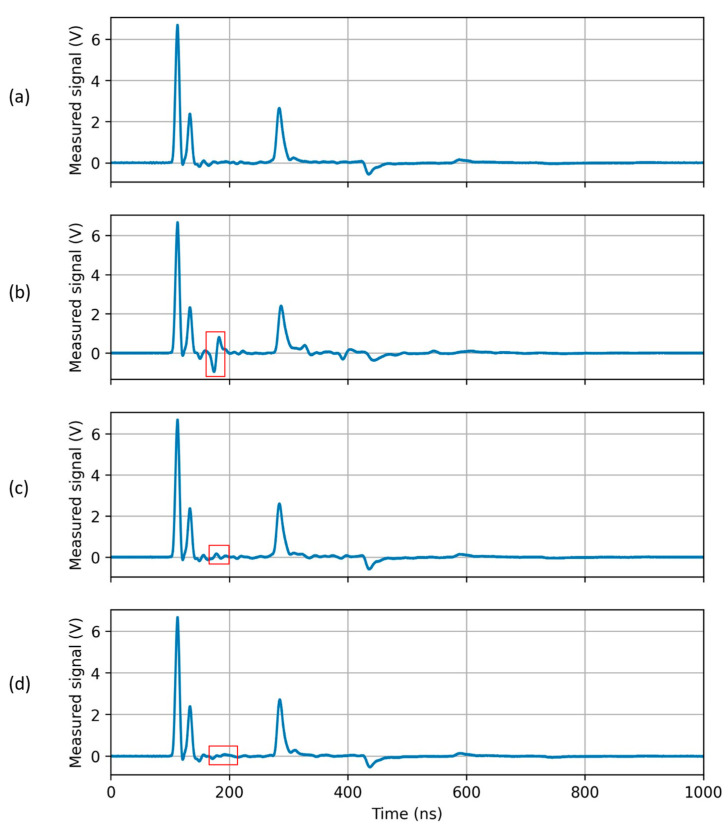
Examples of measured signals, SE length: 15 m. The depicted signals have been obtained in the following conditions: (**a**) SE in absence of water; (**b**) method 1, 33.5 cm long water container; (**c**) method 2, 33.5 cm long water container; (**d**) method 2, 2 m long water container. In each case, the position of the water container is 4 m from the first end of the SE, and the water height is 2 cm in all cases. Reflected pulses corresponding to water-leak conditions are marked with red boxes.

Typical measured signals that are analyzed in the paper are depicted in Figure 4. Signal (a) is acquired in normal (i.e., dry) conditions. It can be a “reference reflectogram”. Signal (b) is acquired with method 1, with a water container length of 33.5 cm (water height is, for this and the other experiments, about 2 cm). The pulse reflected by water, highlighted by a red box, is clearly observed. Signal (c) is acquired with method 2, with the same 33.5 cm long water container. Due to the distance between the SE and the water (separated by the bottom of the 2 mm thick container), the parameters of the TL are less influenced, and, hence, a smaller reflection is produced. The last signal, in Figure 4d, is also acquired with method 2 but, in this case, the water container is 2 m long. Therefore, this case is similar to (c), but the water is distributed in a wider region.

### 2.3. Measurement System Modeling

**Figure 5:**
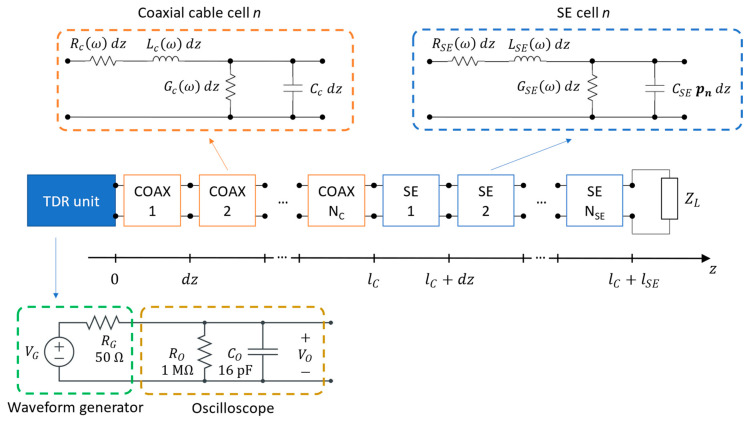
Model of the measurement system.

The first step for locating the water leaks via TDR inversion is modeling the measurement system. The adopted model is represented in Figure 5.

For the coaxial cable and the SE, a classic lumped-element model with pi cells is used, terminated on the load $Z_{L}$. The two TLs are composed of $N_{C}$ and $N_{SE}$ cells, respectively, of length dz. The total lengths of the cables are therefore, respectively,

(1) $$l_{c}=N_{c}\;dz$$

(2) $$l_{SE}=N_{SE}\;dz$$

The following frequency-dependent models are used for the primary parameters R, L, G, C, for both the coaxial cable and the SE:

(3) $$R\left(\omega \right)=R_{0}+R_{1}\sqrt{\omega }$$

(4) $$L\left(\omega \right)=L_{0}+L_{1}/\sqrt{\omega }$$

(5) $$G\left(\omega \right)=G_{0\;}\omega$$

(6) $$C=C_{0}$$

where ω is the angular frequency and $R_{0},\;R_{1},\;L_{0},\;\;L_{1},\;G_{0},\;\;C_{0}$ are six adjustable parameters. The employed functions of the frequency are justified by the dielectric dispersion and the skin effect phenomena. It can be easily verified that, neglecting these effects, the model would be able to reproduce only very coarsely the actual observed reflectograms.

The capacitance of the SE, which is the parameter of interest in the analysis (influenced by the permittivity of the medium around the SE), is independent of the frequency, but dependent on the position z=n⋅dz. Thus, the model of the capacitance per unit length of the n-th cell is

(7) $$C_{0}\left(z\right)=C_{0,n}=C_{SE}\cdot p_{n}$$

where $p_{n}$ is a multiplicative parameter applied the n-th cell. The circuital model is completed by that of the waveform generator and of the oscilloscope, also depicted in Figure 5, and by the impedance $Z_{L}$ of the SE termination.

The described circuital model is clearly only an approximation of the physical reality and is not intended to provide a faithful representation of the electromagnetic behavior of the measurement system, as a full-wave simulation can do. Non-TEM modes and radiative phenomena, for example, are not modeled. However, the model can produce reflectograms that fit on-field measurements very well, as shown in Section 3. Moreover, the model is much simpler and computationally cheaper than full-wave models and can, therefore, be effectively integrated in the simulation-based optimization routine needed for its identification.

### 2.4. Measurement System Simulation

The model is used to actually simulate the measurement system and generate the reflectograms corresponding to given values of all the parameters. To this purpose, it is implemented in the simulator described in [30], which works in the frequency domain and is able to compute efficiently the output, handling frequency-dependent primary parameters. In order to obtain good simulation results, the cell length must be chosen conveniently small with respect to the minimum wavelength $\lambda_{min}$ propagating in the cables. The cell length is given by:

(8) $$\begin{array}{c} dz=\lambda_{min}/\alpha_{\lambda }\\ \lambda_{min}=\min\left\{\underset{f\in \left[0,f_{\max}\right]\;}{\min}\frac{1}{f\sqrt{L_{C}\left(f\right)C_{C}}},\underset{f\in \left[0,f_{\max}\right],z}{\min}\frac{1}{f\sqrt{L_{SE}\left(f\right)C_{SE}\left(z\right)}}\right\} \end{array}$$

where $f_{max}$ is the maximum frequency considered in the simulation, and $\alpha_{\lambda }$ is a tunable parameter. The frequency $f_{max}$ is determined by the impulsive signal of the generator; a rule of thumb for the choice of $\alpha_{\lambda }$ is $\alpha_{\lambda }\geq 10$ [30]. The value of this parameter determines the density of the TL model and, hence, the accuracy in simulating a continuous system. In this work, the value $\alpha_{\lambda }=20$ has been used; greater values lead to negligible improvements and higher computation time. Since during the optimization the values of the parameters and, therefore, of $\lambda_{min}$, change at each iteration, the number of cells is recomputed each time, according to Equations (8), (1) and (2).

### 2.5. Measurement System Identification

The system model is identified by minimizing the root mean square error (RMSE) between the measured reflectogram and the simulated one. Minimizing this objective function is conceptually very simple but requires some attention for its actual implementation.

It is important to underline that, in principle, all the parameters of the model described in Section 2.3 can be involved in the optimization process, including the impedance of the generator and of the oscilloscope. We kept these impedances fixed to their nominal values reported in Figure 5, since optimizing them proved, in our experiments, not useful to improve the fitting. We also kept fixed the length $l_{C}$ of the coaxial cable used for the connection and the length $l_{SE}$ of the SE, which are easy to be known in advance with sufficient accuracy.

It is convenient for the optimization process to introduce a functional representation with a fixed number of parameters of the position-dependent capacitance profile $p_{n}$, since the number of cells depends on $\lambda_{min}$ and is not fixed (besides being quite high, $N_{SE}≃1000$ in our tests). A possibility is the multi-sine signal, which represents well a general band-limited function. In our case, however, we opted for representing $p_{n}$ as a sum of $N_{g}$ equispaced Gaussian pulses, with standard deviation equal to the distance between the peaks of two consecutive pulses, and different amplitudes, which are, therefore, the free parameters. This model is more effective for the optimization process, because each parameter influences a limited region of the profile, and not the whole profile as in the case of the multi-sine signal. It was determined that $N_{g}=100$ parameters are able to describe the capacitance profile with sufficient generality for typical measured reflectograms. This parameter can be increased, however, when working with more irregular reflectograms.

As a final note, the termination impedance $Z_{L}$ is modeled as a capacitance to consider the fringing at the end of the SE. This model proved to lead to better simulation results with respect to an ideal infinite impedance, which causes the presence of a peak in the final part of $p_{n}$ (an effect that clearly compensates the unmodeled fringing capacitance).

The complete list of the free parameters of the model, subject to the optimization process, is listed here.

- Primary parameters of the coaxial cable: $R_{0,c},\;R_{1,c},\;L_{0,c},\;L_{1,c},\;G_{0,c},\;C_{0,c}$.
- Primary parameters of the SE: $R_{0,SE},\;R_{1,SE},\;L_{0,SE},\;L_{1,SE},\;G_{0,SE},\;C_{0,SE}$.
- Vector of 100 amplitudes of the Gaussian pulses composing the capacitance profile $p_{n}$.
- Termination capacitance: $C_{L}$.

Determining and handling an analytical expression of the gradient of the objective function is an unrealistic task due to the complexity of the frequency-dependent model. Given also the large dimensionality of the parameters vector, a random search method with adaptive step size is effectively used for the optimization [31]. Discussing in detail the numerical optimization algorithm is beyond the scope of this work. The results, instead, deserve a careful analysis, presented in the next section.

### 2.6. Water-Leak Detection and Localization

There are two ways to detect and localize water leaks from the estimated capacitance profile $p_{n}$. The first does not use any reference reflectogram but is more prone to errors and ambiguities in interpreting the profile. The second must use the reference reflectogram but gives automatic and univocal results.

When reflections due to the presence of water are strong, like in Figure 4b, water leaks can be directly localized as prominent peaks in the estimated capacitance profile $p_{n}$. We refer conventionally to this procedure as “direct water-leak detection”.

When reflections due to the presence of water are weak, like in Figure 4c,d, the estimated profile does not provide a solid identification of leaks. In these cases, the reference signal measured with the same setup but without water (Figure 4a) is exploited in the following way. We refer conventionally to this procedure as “water-leak detection after calibration”.

1. A first model identification is performed on the reference reflectogram. All the model parameters are determined. This is the calibration phase, which gives the profile $p_{n}=p_{cal}\left(z\right)$ (with z=n⋅dz).
2. A second model identification is performed on measurement reflectogram. In this case, the formerly determined parameters are fixed. The fitting is obtained by acting only on the parameters of an additional capacitance profile $p_{leak}\left(z\right)$, i.e., assuming the capacitance profile $p\left(z\right)=p_{cal}\left(z\right)+p_{leak}\left(z\right)$. This is the measurement phase.

**Figure 6:**
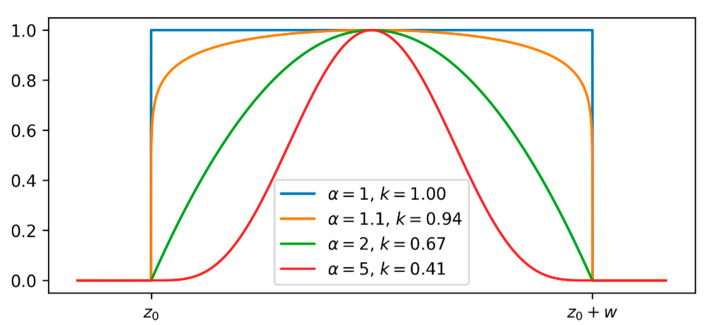
Examples of profiles $p_{leak}\left(z\right)$ obtained using model (9).

The additional capacitance profile associated with a water leak is modeled as a function of the form:

(9) $$p_{leak}\left(z\right)=k\cdot \beta \left(\frac{z-z_{0}}{w},\alpha ,\alpha \right)$$

where β(x,a,b) is the beta probability density function computed in x, with form parameters a and b (see Figure 6). In this way, $p_{leak}\left(z\right)$ is non-zero in the interval $\left[z_{0},z_{0}+w\right]$ (it begins at $z_{0}$ and has a width w); k is a magnitude factor; α is a form factor. Depending on the value of α, model (9) assumes different shapes, from a rectangular pulse to a bell-shaped pulse, as depicted in Figure 6.

## 3. Results

The water-leaks estimation technique has been applied to signals measured with the experimental setup described in Section 2.2.

### 3.1. Direct Water-Leak Detection

**Figure 7:**
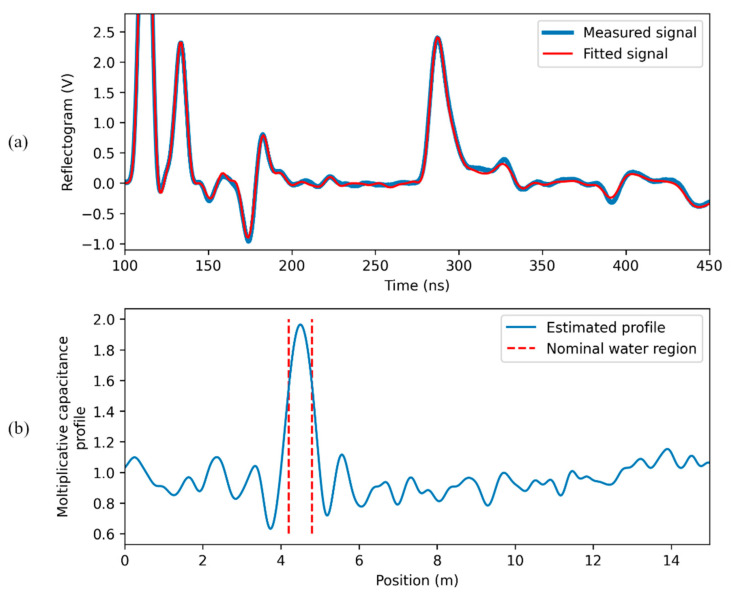
Results of the “direct water-leak estimation” in the case of a strong reflected pulse: (**a**) measured and fitting signal; (**b**) estimated capacitance profile.

When the signal reflected at the water leak is strong (case (b) of Figure 4), the region of the SE submerged in water is accurately identified, as shown in Figure 7.

**Figure 8:**
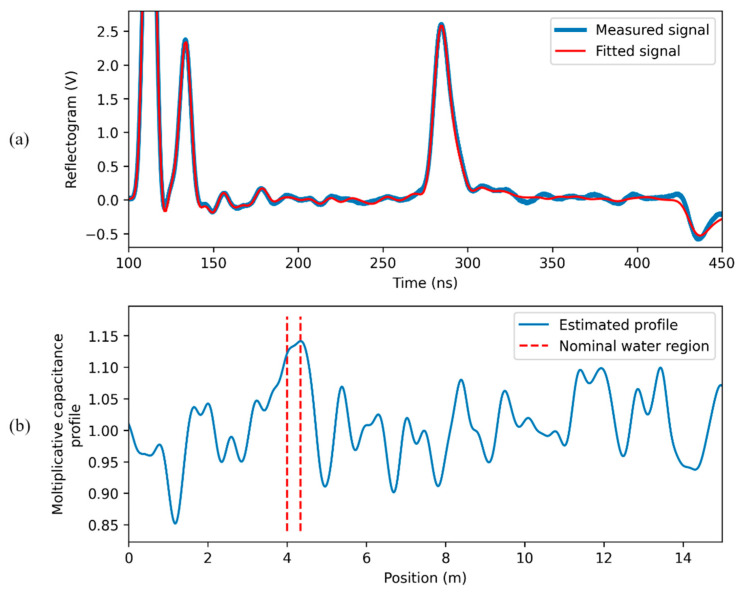
Results of the “direct water leak estimation” in the case of a weak reflected pulse: (**a**) measured and fitting signal; (**b**) estimated capacitance profile.

When the signal reflected at the water leak is weak (case (c) of Figure 4), results are of the kind illustrated in Figure 8. The region where water is present is still recognizable from the fitted capacitance profile, since it corresponds to its highest peak. However, the magnitude of the peak is not so high with respect to the fluctuations in the profile due to the non-idealities of the SE; the detection and the localization are clearly prone to subjective interpretations. In this case, the procedure “water-leak detection after calibration” is justified.

### 3.2. Water Leak Detection after Calibration

#### 3.2.1. Calibration Phase

**Figure 9:**
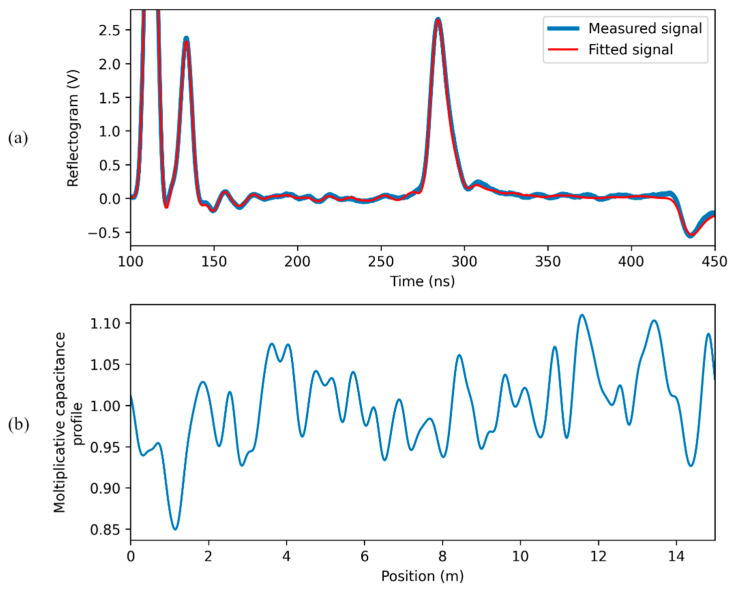
Fitting of the measured signal in the absence of water leaks: (**a**) measured and fitting signal; (**b**) estimated capacitance profile.

The result of the calibration phase of the procedure is shown in Figure 9. The figure shows that the obtained fitting follows the uneven oscillations of the measured reflectogram very well, even after the principal reflected pulse (i.e., even multiple reflections are reproduced quite well). It is important, however, to point out that the estimated profile must not be interpreted as the actual physical capacitance profile along the SE. The model greatly simplifies the electromagnetic behavior of the system, and, even more important, the transmitted pulse is quite large: to accurately estimate the capacitance profile, a greater spatial resolution is needed, and, hence, a shorter transmitted pulse must be used (this kind of measurement needs costly instrumentation not suitable for on-field use; it has been obtained in some preliminary tests, not of interest here).

The obtained profile, therefore, must be interpreted simply as one of the profiles that reproduces well the input/output behavior of the system. This is sufficient, however, to detect and locate quite accurately the water leak.

#### 3.2.2. Measurement Phase—Case (b)

**Figure 10:**
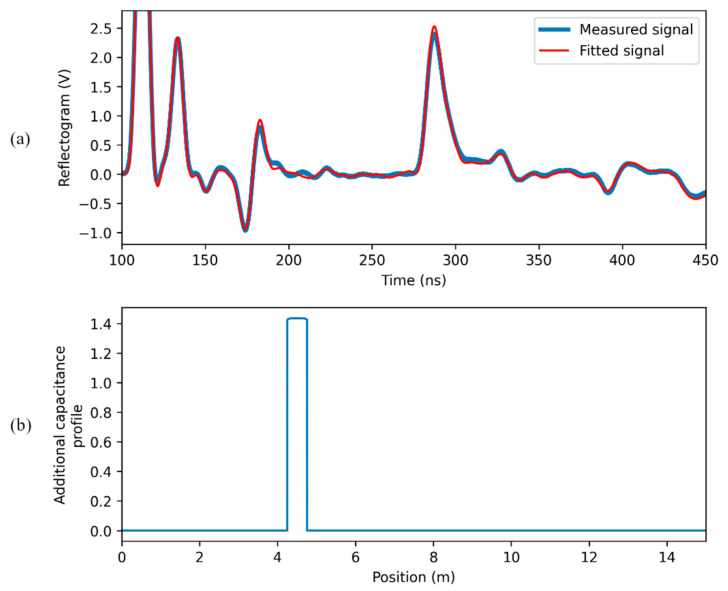
Results of the measurement phase for the reflectogram of Figure 7: (**a**) measured and fitting signal; (**b**) estimated additional capacitance profile.

Using the calibration described above, the measurement phase of the procedure “water-leak detection after calibration”, as described in 2.6, is applied to the reflectogram of Figure 7, obtaining the results reported in Figure 10. The estimated center and length of the water region are 4.50 m and 51 cm, respectively, while the actual ones are 4.52 m and 46 cm, respectively.

#### 3.2.3. Measurement Phase—Case (c)

**Figure 11:**
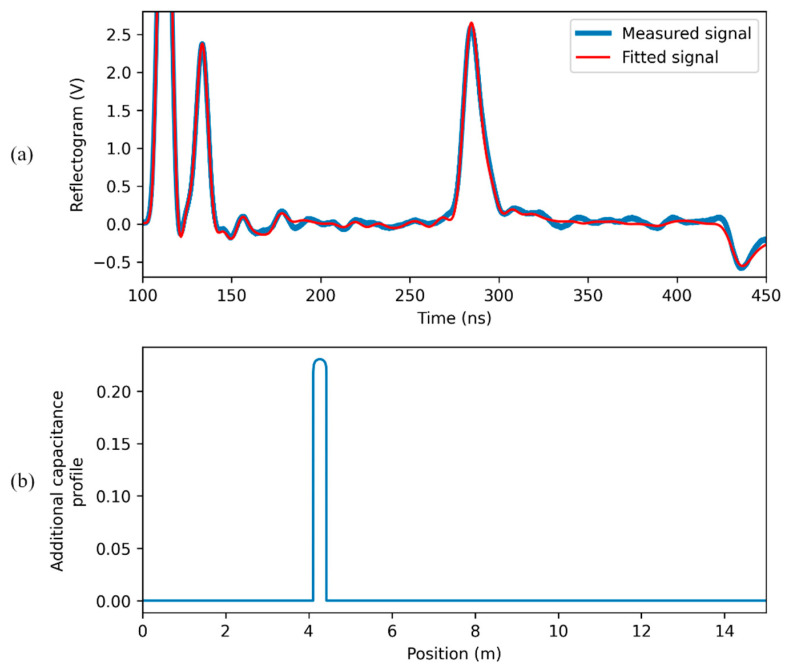
Results of the measurement phase for the reflectogram of Figure 8: (**a**) measured and fitting signal; (**b**) estimated additional capacitance profile.

Using the same calibration, the measurement phase is applied to the reflectogram of Figure 8. The results are depicted in Figure 11. Despite the very small reflection due to the water, the water region is correctly and accurately identified.

The same test is repeated by placing the water container in other positions, and results are reported in Table 1. The estimated water region always intersects the actual water region, which can be classified as a successful localization. The standard deviation of the error in the leak-center estimation and that in the leak-length estimation are both approximatively equal to 13 cm. In Table 1 is also reported the total estimated capacitance of the leak, i.e., the area below the estimated additional capacitance profile. This figure can be useful as an index of the size of the leak, in terms of quantity of water sensed by the SE.

**Figure 12:**
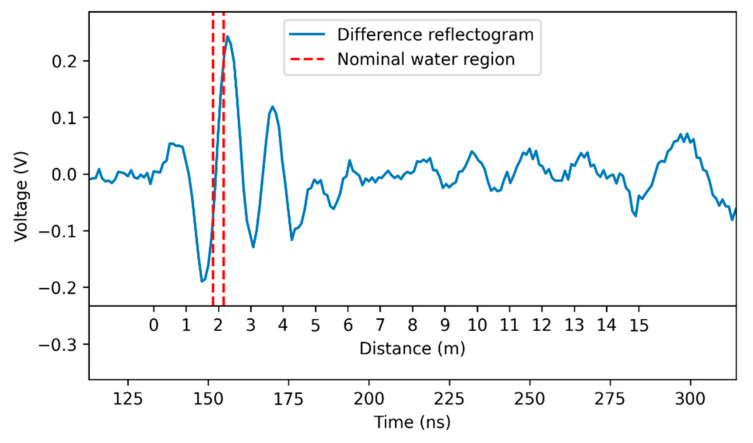
Difference between the reflectogram measured in the presence of water (Figure 11) and the reference reflectogram (Figure 9). The indicative distance scale depicted in the Figure starts from the position of the pulse reflected at the coaxial/SE interface and ends at the position of the pulse reflected at the SE termination.

The signal in Figure 11 has also been analyzed through traditional water leak detection techniques, for comparison. Traditional techniques are mainly based on direct observation of the measured reflectogram which leads to the identification of reflected signals. In this case, in which the reflected pulse is very small, the “difference reflectogram” between the measurement reflectogram and the reference reflectogram (depicted in Figure 9) is more informative. The difference reflectogram has been, therefore, computed and is depicted in Figure 12. The Figure also contains an indicative distance scale that correlates time instants to distances from the beginning of the SE. The origin of the scale corresponds to the peak of the pulse reflected at the coaxial/SE interface, while the length of the SE (15 m) corresponds to the peak of the pulse reflected at SE termination. In the difference reflectogram, the reflection caused by the presence of water is clearly visible. Oscillations in the signal are, however, present in the whole region from ~1 m to ~5 m, making it difficult to accurately localize the leak and reducing, therefore, the practical applicability of the method.

#### 3.2.4. Measurement Phase—Case (d)

Water leak detection is then carried out for 2 m long simulated leaks, i.e., case (d) described in Section 2.2 and in Figure 4; the calibration is that of Section 3.2.1. The results are reported in Table 2. In this case, water leaks are correctly localized in each measurement (estimated and actual water regions always intersect). The standard deviations of the error in the leak center estimation and in the leak length estimation are approximately equal to 33 cm and 43 cm, respectively.

## 4. Conclusions

In this paper, a TDR inversion method for enhanced TDR-based water-leak detection and localization is proposed. The detection and localization of water leaks is performed by processing reflectograms taken on a bi-wire sensing element placed alongside the buried pipes. TDR inversion uses a model designed to reproduce accurately the uneven and oscillating reflectograms obtained in a leak detection campaign and to be identified even if very little prior information on the model parameters is available, as is unavoidable in this particular kind of measurements. The TDR inversion can localize water leaks working on a single signal, when the reflection caused by the water is strong enough, and exploits an initial calibration carried out in the absence of water, in case of weaker reflections. Experimental results carried out in different conditions show that the method is able to produce accurate estimates of the position and the extension of water leaks. The proposed method has, therefore, the potential to be used successfully also for continuous monitoring of water pipes and has the potential of being employed for other TDR-based applications, since it basically is able to perform a TDR inversion with no prior characterization of the TDR probes and of other components of the measurement system.

## Figures and Tables

**Table 1:** Estimation results for the water leak position and entity estimation in the case of leak length 33.5 cm.

Nominal Center of Water Leak (m)	Measured Center of Water Leak (m)	Measured Length of Water Leak (cm)	Measured Total Capacity (% of First Measure)
2	1.97	33.2	-
4	4.11	21.6	64.40
6	6.02	30.4	109.63
8	8.14	19.8	77.54
10	10.41	62.2	136.93
12	12.25	22.0	109.95
14	14.14	37.5	117.07

**Table 2:** Estimation results for the water leak position and entity estimation in the case of leak length 2 m.

Nominal Position of Water Leak (m)	Measured Position of Water Leak (m)	Measured Length of Water Leak (cm)	Measured Total Capacity (% of First Measure)
2	1.64	132.20	-
4	3.59	114.56	86.85
6	6.22	183.06	211.06
8	7.90	241.84	187.61
10	10.60	118.45	121.66
12	12.22	124.75	182.85
14	14.18	147.00	115.07

## Data Availability

The data presented in this study are available on request from the corresponding author.
